# Integrating artificial intelligence in pathology: a qualitative interview study of users’ experiences and expectations

**DOI:** 10.1038/s41379-022-01123-6

**Published:** 2022-08-04

**Authors:** Jojanneke Drogt, Megan Milota, Shoko Vos, Annelien Bredenoord, Karin Jongsma

**Affiliations:** 1 Department of Medical Humanities, University Medical Center, Utrecht, The Netherlands; 2 Department of Pathology, Radboud University Medical Center, Nijmegen, The Netherlands

**Keywords:** Medical ethics, Medical imaging, Pathology

## Abstract

Recent progress in the development of artificial intelligence (AI) has sparked enthusiasm for its potential use in pathology. As pathology labs are currently starting to shift their focus towards AI implementation, a better understanding how AI tools can be optimally aligned with the medical and social context of pathology daily practice is urgently needed. Strikingly, studies often fail to mention the ways in which AI tools should be integrated in the decision-making processes of pathologists, nor do they address how this can be achieved in an ethically sound way. Moreover, the perspectives of pathologists and other professionals within pathology concerning the integration of AI within pathology remains an underreported topic. This article aims to fill this gap in the literature and presents the first in-depth interview study in which professionals’ perspectives on the possibilities, conditions and prerequisites of AI integration in pathology are explicated. The results of this study have led to the formulation of three concrete recommendations to support AI integration, namely: (1) foster a pragmatic attitude toward AI development, (2) provide task-sensitive information and training to health care professionals working in pathology departments and (3) take time to reflect upon users’ changing roles and responsibilities.

## Introduction

Artificial Intelligence (AI) technologies are increasingly being developed for image-based diagnostics. For pathology, these technologies promise to support pathologists in time-consuming and repetitive tasks^1,2^ and may also move the field forward towards new knowledge, discoveries and “breakthroughs”^1,3^. Such algorithm-based technologies have been developed for metastases detection, Ki67 scoring, tumor-infiltrating lymphocyte (TIL) scoring and Gleason grading, as well as in predicting the status of molecular markers on HE slides^1^. The development of AI is even seen by some scholars as a potential “revolution” of the field, since AI could provide pathology with substantial new knowledge and new ways of operating^1,4^. Whether or not the implementation of AI in the pathologist’s diagnostic process will indeed cause a revolution^5^, it will likely herald changes in image-based diagnosis^1–10^.

The implementation of AI within pathology is dependent on a successful digital transition, where departments shift from using traditional light microscopes to assessing digitalized tissue slides on a computer screen^6–9^. This transition can positively impact the pathologist’s daily workflow. For instance, digital images can be stored and quickly accessed in a digital archive; this makes it possible to easily consult colleagues remotely^6,7^. There are also several challenges to the adoption of AI-based applications in pathology when looking at the current state of digital pathology. For instance, images can currently be digitized in a 2D format, yet fast and high-quality 3D imaging is still largely untenable – meaning some diagnostic tasks (e.g. cytology) that require 3D images must still be conducted with a microscope^10^. Furthermore, conditions for developing AI technologies that can analyze a diverse range of images are not yet optimal. For instance, most AI algorithms require “labelling” (i.e. annotation) by a pathologist, preferably an expert, who manually delineates the area of interest (i.e. anomaly or malignancy) by which the algorithm can be trained^6^. Because of time constraints and the financial burden of labelling, systematically expert-annotated images are still scarce. Also, long-term storage of digital images for potential future development requires large and costly data storage facilities. This can therefore constitute a roadblock for pathology departments wanting to digitalize their workflows and needing large data sets for training AI. Finally, pathology images of several basic types of tissue are characterized by a pervasive variability of patterns, which can make comparisons across large data sets more difficult^6^.

Despite these challenges, AI can still constitute a highly impactful technology. Scholars have therefore recommended involving pathologists in development, implementation, and governance processes in order to optimize this impact^2,6,11^. Previous empirical studies – two surveys and two qualitative interview studies – have focused on exploring the views of pathologists concerning AI^12–15^. These empirical studies have thus far focused on general attitudes towards AI. They have concluded that pathologists are overall positively inclined towards AI. These studies also indicate how necessary – and poorly understood – pathologists’ views and insights are concerning AI’s place within their field^13^. There is still little known, for instance, about the perspectives of pathologists concerning the current and future integration of AI within their daily work and how responsibility should be approached in the implementation of AI within the diagnostic process.

The current study aims to fill this gap in knowledge and presents the results from the first in-depth interview study, as far as we are aware, on the integration of AI within pathology. In addition to gaining insight into the professionals’ stance towards possibilities for AI integration, our goal was to analyze their views in connection to the broader social and ethical context of AI development. In this article, we will focus primarily on the issue of responsibility. First, we will describe pathologists’ views concerning possibilities, prerequisites and conditions for AI integration; we will then situate these views within the broader context of AI implementation. Finally, we will formulate three concrete recommendations to support successful AI implementation in the clinical decision-making processes of pathologists.

## Materials and methods

We conducted a qualitative interview study to investigate the perspectives of pathologists on the development and implementation of AI. The study design is in accordance with the consolidated criteria for reporting qualitative studies (COREQ)^16^. We have opted for qualitative methodology, and specifically semi-structured interviews, since these are particularly suited to investigate complex phenomena, such as AI, encountered in health care practices by focusing on elucidating different perspectives^16^. By adopting this kind of methodology, the study is able contribute to our understanding of AI’s potential impact on the work of pathologists, lab technicians and computer scientists, and could help us apply their perspectives to future AI systems within pathology.

### Research design

This study constitutes part of the Responsible Artificial Intelligence in Clinical DecisIOn making (RAIDIO) study. In order to gain insight in the integration of AI within the decision-making processes of pathologists and other professionals working in pathology labs, we conducted an inductive qualitative analysis of recorded conversations with pathologists, lab technicians, and computer scientists^17–20^.

### Sampling and data collection

For this analysis we interviewed professionals working at the pathology departments of the UMC Utrecht and the Radboudumc in the Netherlands. These sites were chosen because they had both completed the transition to a primarily digital workspace^21,22^. This made it possible to talk with professionals on the impending integration of AI within their work practices, as well as their hopes and expectations concerning AI’s functioning. Both institutions have their own computer science teams and collaborate with different external parties on AI development, whereby the AI applications being proposed, implemented, or fine-tuned at each of these sites vary in the role they play in the decision-making process. We therefore expected that combining both institutions in this study would result in a rich variety of perspectives on the potential use of AI in pathology.

Interviews were conducted between June 2020 and February 2021. Because of the pandemic, the conversations were conducted via telephone; JD and MM conducted interviews both individually and as a team. A semi-structured topic list was used to guide the conversations. The recorded interviews were transcribed verbatim by a professional transcription service and checked for reliability by JD. The transcripts were then coded for confidentiality and identifying information was removed. The interviewees were invited to perform a member check of their own transcript. The interviews were conducted in Dutch and translated to English by JD and MM.

### Data analysis

The data selection and analysis occurred inductively and iteratively^23^ by means of constant comparison^24^. The software program NVivo12 supported the data analysis. JD and MM read individual interview transcripts and independently identifying conversation fragments, or units of meaning^17–20^ they considered relevant to the research question; after each interview they met to compare their observations. After four interviews, they began grouping these fragments into descriptive categories, resulting in the first code tree. They then discussed this code tree with other members of the research team (KJ, SV, and AB) as a means of further refining the code tree. Next, JD and MM sampled and independently coded 15 transcripts. These independent coding results were compared multiple times and discussed as a means of further refining the code tree. JD then coded the remaining transcripts, adjusting the code tree when necessary. Finally, MM and JD performed an intercoder reliability check by recoding four transcripts (2 pathologists, 1 lab technician, and 1 computer scientist) and comparing their results. This final step also served as a means of checking for meaning saturation^25^.

### Data statement

The data has been presented by means of illustrative quotes, which were carefully selected to represent the arguments presented in the interviews and do justice to the variety of perspectives shown within them. In the selection, we have also considered whether the quotes could be understood without the context in which they were originally uttered. The complete datasets themselves are not publicly available because the individual privacy of the participants could be compromised. The individual privacy of the participants particularly important as their statements included opinions and beliefs regarding the ways in which AI should be adopted. These are deemed sensitive therefore fall under the protection of the General Data Protection Regulation (GDPR: article 9).

### Ethical considerations

Ethical approval for the RAIDIO study was obtained from the Medical Research Ethics Committee (MREC) of the University Medical Center Utrecht and Radboudumc (WAG/mb/20/014090). The MRECs determined that this study was exempt from the Medical Research Involving Humans Act. Written informed consent was obtained from all participating respondents.

## Results

In this study, 45 professionals were invited to participate by means of a department-wide email. Additionally, some professionals were directly approached by either the research team or a contact person at the department to aim for a representative group of professionals with a mixture of experienced pathologists and pathologists in training, and professionals with an active or more passive role in digitalization and AI development. 24 responded to our messages and were interviewed (15 pathologists, 7 lab technicians, 2 computer scientists) (Table 1). The interviews provided a varied sample of perspectives concerning the transition to digital pathology and the possibility of AI-based image analysis.

During the interviews, respondents jumped back and forth between two aspects of the digitalization process. They reflected upon their departments’ recent digitalization processes. They also discussed the potential value AI might have for the future of pathology as a field. Regarding digitalization, multiple respondents described how digital pathology had already significantly advanced and improved their field by increasing time efficiency, facilitating easier communication and the fact that, unlike physical slides, digital images could not be misplaced or accidently switched. At the same time, respondents also described technical challenges when using whole-slide imaging (WSI) to analyze tissue samples. For example, the digital screen cannot always display images at the same level of definition as a microscope, nor can the current scanners create high quality 3D digital images fast enough for cytology specimens. Moreover, specialists working with larger tissue samples mentioned that the digital image could not be viewed in detail in its entirety on the screen; it therefore took them longer to assess such samples accurately.

Similarly, responses about the potential value of AI took on many forms. AI was used within the interviews as an umbrella term for image recognition tasks, other automated tasks, applications based on machine learning or deep learning, but also “simpler” algorithms and calculations. The picture that emerges from the interviews is of AI as a rather amorphous entity, which reflects the ambiguity of the term in broader scholarly and popular discourse. This variety of definitions and understandings could be due, in part, to the fact that not all of the participants were directly involved in AI development. Most participants only knew of the AI tools expected to be integrated in the short-term [see^26^] and had to speculate on the longer-term applications and possibilities of AI. In doing so, the interviewees seemed to draw on their experiences with currently developed tools and digital pathology developments to talk about expectations about future AI-applications.

In following sections, we will further explicate how participants viewed the emergence of AI within the field of pathology, specifically themes related to the future roles and possibilities of AI. In order to illustrate how respondents view AI’s future place in pathology and connect it to the current digital developments, we have identified four themes related to the potential value of AI: (1) prerequisites and considerations for AI integration, (2) AI in the daily workflow, (3) envisioned roles and responsibilities for AI and (4) envisioned roles and responsibilities for pathologists. The interview extracts referred to in the body of text and can be found in Tables 1–4.

**Table 1:** Background characteristics participants.

	*N* (%)
*N* respondents	24 (100%)
Place of employment:	
UMC Utrecht	11 (46%)
Radboud MC	12 (50%)
Both	1 (4%)
Function:	
Pathologist	15 (63%)
Lab technician	7 (29%)
ICT	2 (8%)
Main area of expertise (pathologists):	
Oncology	4 (27%)
Inflammatory disease	4 (27%)
Both	7 (46%)
Experience working in field:	
5–15 years	8 (33%)
More than 15	13 (54%)
Unknown	3 (13%)
Experience working with AI:	
Yes, development and/or validation	12 (50%)
No, development and/or validation	10 (42%)
Unknown	2 (8%)

**Table 2:** Illustrative quotes for theme 1.

	(Sub)Theme	Quote
1	Prerequisites and considerations for AI integration	
1A	From digital pathology to AI implementation	CP 1: If you would ask me now: in how many pathology labs in the world do they have at least one AI tool implemented? Then I would say: zero! (…) AI implementation depends on several premises. If you would like to seriously apply AI, then you must have a completely digital workflow in place. Well, there are only a few labs in the world which have this. Still, labs are slowly making the transition, I believe more will follow, yet at this moment it is still very limited. (…) Secondly, technically speaking, the implementation is much harder than we first thought. (…) An average lab does not have the capacity. We have IT staff; one is specialized in AI implementation. However, how many labs have this luxury? (…) As we do not have that much development power, we have re-directed our focus towards implementation.
1B	The potential impact of digitalization and AI on pathology	CP 19: With digital pathology, you actually have a technology with which you can remove the walls in pathology, make them invisible (…) Decompartmentalizing pathology, and thereby expertise, means that the distance to expertise does not exist anymore. First, expertise can be shared on a national level, and in the future also internationally. If you think about the development of the field and communicating the best information to the patient, this will have a very large impact. This is the possible impact of a digital image, but if you once have a digital image, then you can also develop an artificial intelligence arsenal and a whole new area of development within pathology (…) So, you have a practical side of the current digitalization, with the decompartmentalization, as well as progress in terms of AI development, two things which will have real impact.
1C	The importance of patient care and monetary considerations for AI development	DS 16: It is very easy, and for researchers very fulfilling, to show how great AI is in scientific publications. (…) If you examine these results and think about the real value and who is going to pay for the added value, then AI proves to be disappointing. (…) You can claim that AI is more efficient, that a pathologist with an algorithm is faster than one without. However, there are almost no studies which prove this. (…) So, efficiency is just a possible gain. Another claim is an improvement in the quality of your diagnosis. (…) But how can you show how much better the diagnosis becomes? What does such an improvement mean for the patient? (…) If you show that a pathologist with an algorithm performs somewhat better than a pathologist without, who is going to pay for it? (…) This seems trivial, we as normal people, citizens, potential patients, would always want a better diagnosis. Yet insurance companies are not so keen to pay for it, they argue that the cost [of AI] is an internal concern for hospitals.
1D	AI in relation to broader technological innovation within pathology	CP 17: Over time, you become more hesitant about the success of various developments. When I started working as a pathologist, the electron microscope had just been discovered, which allowed us to very accurately see and recognize all kinds of small parts within cells (…) Well, the electron microscope has indeed led to many insights, yet – at this moment – there are few diseases where you need an electron microscope. This means that the electron microscope has become almost obsolete for patient care. After [the electron microscope], DNA research became more common. (…) This has also provided us with all kinds of insights. However, it was not the egg of Columbus. With artificial intelligence we will probably see a same phenomenon happen: we’ll learn much from it, but it will cause new problems we can’t yet foresee, illustrating that everything is more complicated than we thought.
1E	AI’s importance is dependent on the pathological (sub)specialism	CP 3: For some, an AI tool will be used much more often and will be more important than for other sub-specialisms. Look, I am specialized in kidneys, and therefore inflammations. I think that AI is less applicable for me than oncology, where you have real tumors. (…) AI use is dependent on your area of expertise.

**Table 3:** Illustrative quotes for theme 2.

	(Sub)theme	Quote
2	AI in the daily workflow	
2A	AI to support with the pre-screening of medical images	CP 14: We do a fair amount of looking at the same kind of patterns, and this is a bit like working in a monastery. It costs me little intellectually – it mainly costs time and requires concentration. But it is not a great mental achievement. A computer could do it as well. AI could do it. For example, an AI-algorithm could just indicate on certain slides, ‘look at this one, I suspect there are tumor cells’, and then I could look at that area and say ‘yes, indeed, I also think it’s a tumor’. AI screens it so you don’t have to look at the rest of the sample. (…) This could free up some spare time for us to perform other useful tasks.
2B	Distributing routine tasks within pathology	CP 13: With artificial intelligence, I mainly think about easy tasks. For instance, part of my work is very complex, and part is very easy. All the tasks which are very easy, those moments in which I am diagnosing basal cell carcinomas, for instance, I think ‘did I really study this long to be able to perform this task?’. It would be very nice if such tasks could be completely automated. You would only have to push a button as pathologist, at least in the beginning. Perhaps it could become completely automated at a certain moment. That would be ideal. (…) Basal cell carcinomas are, for me, the greatest bulk task. We have calculated that it takes up one-fifth, or around twenty to twenty-five percent, of our total time. (…) AI could give us more time.
2C	AI to make up the initial report on a medical case	CP 7: Yes, of course, diagnoses that are easy for us, but take time, would benefit from a provisional diagnosis and report. Especially reports. People often speak about image recognition, yet this isn’t really necessary. If I make a simple diagnosis, it costs me a second or less, if it’s nothing special. Writing the report, however, is a time-consuming task, this takes me a couple of minutes. So, everyone is talking about image recognition, but I have the feeling almost nobody thinks about the real value of AI, so to speak.
2D	Integration of different sources of knowledge	CP 10: A problem in pathology diagnostics is the complexity. You have many different possibilities to investigate in most cases; these investigations are often performed in different labs. You also need to integrate clinical data to reach a diagnosis. This requires a lot of coordination and cooperation. And, yes, I can imagine that one possible role for AI could be to collect all the necessary information. Currently, we do this ourselves. (…) In this way, all the various techniques, and the results of the investigations--which are not the same as the images themselves-- could be brought together and perhaps even partly integrated.
2E	AI to make pathology more evidence-based	DS 16: AI algorithms have the potential to make pathology more consistent and much more evidence-based. Also, AI could help a larger number of patients receive the most optimal treatment. I think there is much value to be found in AI.
2F	Learning from an AI tool	CP 5: I hope it will be possible to ask, ‘why do you say this?’ if you have a question about an AI outcome. And, ‘why is it not this and why not that’, and that you receive an answer. This will probably happen, which means we can learn a lot from AI. And we will probably learn more from it than a pathologist who speaks from experience and says ‘yes, but I just see this.’
2G	AI to standardize pathological processes	A 21: Pathology will always depend on interpretation, because we work with tissue samples; the tissue of patient A is never the same as the tissue of patient B. But we try to standardize all steps leading up to a diagnosis as much as possible. If lab technician A does it, it will be a little bit different than when lab technician B does it. And you want to reduce the variation. A technological device could help. This doesn’t mean replacing employees. Employees will always be needed to manage the functioning of such technologies. They will need to be there to interpret why a technology doesn’t perform its duty and how failures happen. But you do want to standardize as much as possible.
2H	AI to standardize image recognition	CP 11: A computer does not get tired and (…) looks at the sample the same way every single time. This means it’s more standardized. If AI can learn to recognize a tumor based on the number of annotations of experts or immune colorings, then it is probably possible for it to find a more objective means of diagnosing tumors than pathologists.
2I	AI to support in discussions between pathologists	CP 6: AI algorithms are in principle based on pathologists. That’s why I am not of the opinion that an AI algorithm is 100 percent objective. It could be of added value when pathologists disagree, as it goes beyond the opinion of one individual. So it could help in a discussion. But it might just make things more complicated, the more knowledge we have the more complex diagnostics becomes. (…) I don’t think objectivity is really something we should strive for. I do think it’s a good thing for pathologists to become more unified in the future.

**Table 4:** Illustrative quotes for theme 4.

	(Sub)theme	Quote
4	Envisioned roles and responsibilities for pathologists	
4A	AI can take variety of potential roles, yet it can be overruled	CP 1: These algorithms are meant to support our work, to make it better, to make it faster, to make it more fun, to make it less tedious, to make it more reproducible. But this doesn’t imply that we won’t look at slides anymore. Every output of an algorithm will be checked by us. We will overrule it if we do not agree.
4B	AI as an autopilot function	CP 14: I always compare the possibility of AI in pathology to aviation. AI is like the autopilot. The pathologist is responsible for the final diagnosis, but he or she uses tools to arrive at this diagnosis. Such a tool can save time by performing certain work, certain tasks within pathology. And yes, this is a kind of autopilot-phenomenon. So, you as pathologist hold final responsibility for the diagnosis, but you are supported, in part, by a computer.
4C	AI as a machine in clinical chemistry	CP 3: I think the pathologist should remain responsible. (…) You can compare it with clinical chemistry. The clinical chemist remains responsible for the way machines function. You cannot blame the machine when it is not correctly calibrated. The pathologist should stay responsible for the diagnosis, but also that the technique functions as it should. So, if he doubts an AI output, then he should be able to say: ‘no we won’t use this, because I don’t trust it.’
4D	Trusting algorithmic outcomes by means of reproducibility	CP 19: One of the concerns people raise, is how pathologists can take responsibility for the application of a black box technique, something that uses deep learning. How can I take responsibility for the results from such a tool? (…) My first response is, look, I have a telephone and I have no idea how it works, but I do know how to use it, and that is sufficient grounds for me to trust it as a reliable technology, something I can depend upon it, with reproducible results. So, you do not necessarily have to understand how something works to trust it.
4E	Technical details are less relevant to diagnostic processes	CP 9: I know the basic patterns that determine how the system [AI] works. I can explain a little bit about how it works, but I understand nothing of the details, This is too technical for me, but – if I am honest – I don’t consider this important. I must have a basic understanding how it works, what the basic principles are, but I don’t have to know the details of the technological functioning. If I can see how the technology works. It’s just like a telephone. We all use a telephone. I have no idea how the thing works. I don’t have a problem with that. I think you should know the basic principles [of AI], otherwise you can’t understand how it arrives at a certain diagnosis, but I don’t have to know every detail. It’s a matter of checking whether it is the right diagnosis.
4F	Black boxes are everywhere in our daily lives	DS 16: People ask: ‘will it replace us?’, ‘What does it mean for our work?’ And they say ‘I don’t trust this black box’. The black box is a large fear factor. I must honestly say, I do not believe in this kind of sentiment, I think we allow many black boxes in our lives. The fear of the unknown leads us to believe we need some sort of understanding without really needing it. I think we do many things within pathology, in medicine, but also outside of medicine, when we have no idea what’s happening. You get into your car, you have no idea what happens inside of it. You don’t have a clue. Especially modern cars, with all the software and computer programs.
4G	Explainability becomes important in more complex AI	DS 12: Say you have a model that provides a prognosis or diagnosis (…) and it says ‘this is cancer’. Then you’ll want to know, (…) how did the algorithm reach this conclusion? But for simple things like mitosis, I think it’s less relevant. This is also the topic of explainable AI research, explaining how algorithms are made, how do they reach certain results, what are the features the algorithm deems important or interesting, how does it make a choice. Well, I don’t think such concerns are applicable now, but if AI becomes more complicated in the future and we depend more on it, then maybe such concerns will become relevant.
4H	Pathologists need to know how AI is designed to take responsibility for algorithmic mistakes	CP 20: They don’t have to know everything about the technical programming, but they must know how people designed AI. Because if something goes wrong, then it might be easier for a pathologist to trace back where it went wrong.

### Prerequisites and considerations for AI integration

When reflecting on the potential use of AI within digital pathology, four categories of consideration have been identified in the comments of respondents. First, their responses substantiated the intrinsic relation between digital pathology interfaces and AI. As a digital workflow is necessary for implementing AI in the decision-making process of a pathologist (Table 2, Quote 1A), AI is dependent on the extent in which a pathology lab is digitalized. Furthermore, the quality of digitalized scans impacts the possibility to train and validate new AI applications. Respondents also described the value of digital pathology and AI as being closely intertwined. As one respondent explained, digital pathology enables pathologists to share medical images – along with medical expertise – nationally and internationally (Table 2, Quote 1B). Similarly, if digital archives are created, AI can be implemented to analyze the images on a larger scale. This means that the combination of digital pathology and AI could result in a broadening of medical expertise and, at the same time, open up new means of acquiring knowledge.

Second, although they also believed in the great potential of AI for pathology, some respondents addressed the fact that the implementation of AI applications within their departments will likely be determined by the application’s ultimate contribution to patient care combined with the costs of developing or purchasing such applications. Both pathologists and computer scientists reported that – despite the great promises of AI as reported in the literature and at conferences – an application’s value depends on the measurable improvements (e.g., in effectivity or in better diagnostics) of the implementation and who is willing to pay for these improvements (Table 2, Quote 1C). Practical feasibility therefore constitutes a key component of successful AI development according to several professionals.

Third, when reflecting on the possible impact of AI on pathology, some respondents emphasized the importance of maintaining a realistic stance towards the potential value of AI. This reserved stance stemmed from their experience and (sub)specialism within the field. For instance, when compared with earlier technological innovations in pathology, AI may be ‘just’ another step towards understanding the complexity of the human body. Respondents also compared the current hype around AI to previous technologies that promised to fundamentally change the field. The electron microscope and DNA research are two examples cited within the interviews. These innovations have contributed to advancements in the field, but they have also resulted in more complex knowledge on the ways in which disease mechanisms work and can therefore make interpretation of clinical cases even more difficult (Table 2, Quote 1D). Furthermore, AI applications may not be relevant to all (sub)specialisms within pathology (Table 2, Quote 1E). Many respondents also emphasized that a large part of their work is integrating and interpreting information from a diverse range of sources (such as tissue samples, histological images and molecular data) into a diagnosis. Their relativizing views on AI development are hence guided by the already highly technical nature of their work.

**Fig. 1:**
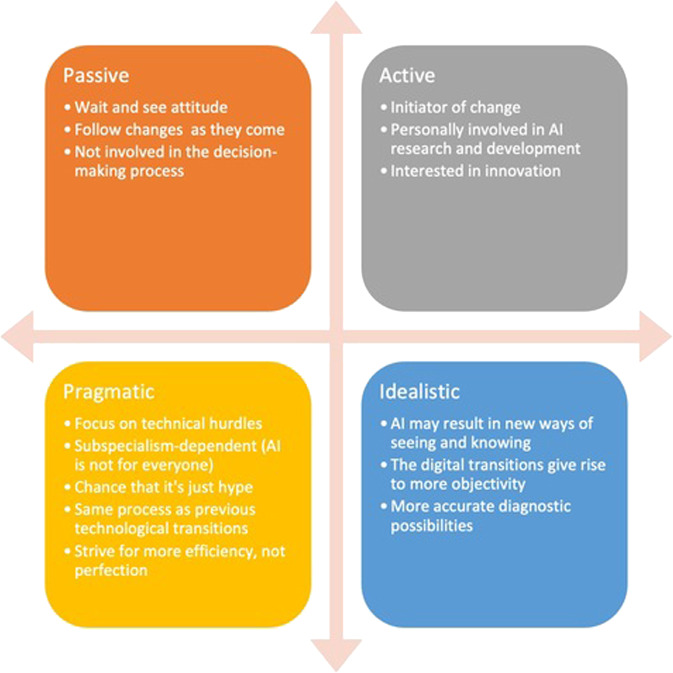
Positioning of respondents concerning digital transitions.

Fourth, we found that respondents either took a passive or active stance towards the digital transitions and potential AI applications. As Fig. 1 illustrates, a passive stance was often accompanied by a ‘wait and see’ attitude and was mainly adopted when the respondent was not involved in AI development or not able to make executive decisions concerning its future implementation. Respondents showed an active attitude towards AI when they were interested in innovation, initiated it within their departments, or were personally involved in AI research and development. Similarly, when discussing the possible consequences of AI implementation, respondents showed either a more idealistic or pragmatic perspective towards the future. Idealistic perspectives focused only on the promises and benefits of AI for pathology, while pragmatic perspectives focused on the benefits of AI as well as important hurdles to AI development and implementation. Moreover, a distinguishing feature of the respondents was whether they mainly worked in the context of oncological or inflammatory diseases. The promise of AI seems to be more apparent in diagnosing oncological diseases^1,27^ than inflammatory diseases, which could explain the more optimistic stance towards AI development amongst by respondents mainly working with oncological tissue samples.

### AI in the daily workflow

Respondents often had clear and specific hopes and expectations for AI with regard to their daily tasks and workflow. Overall, respondents were almost unanimous in their expectation that AI would increase the efficiency of their workflows. Efficiency is of great relevance in pathology, a point respondents repeatedly underscored. For example, one pathologist remarked that their time is costly, and is therefore best spent on complex cases. For some respondents, the ideal AI application would be one that could complete or support simple, routine, or repetitive tasks. Examples frequently mentioned by respondents were counting mitoses in a digital image or diagnosing basal cell carcinoma. These kinds of tasks are reportedly not intellectually taxing (Table 3, Quote 2A) and can take up around 20 to 25 percent of a pathologist’s time (Table 3, Quote 2B). A few respondents made a different argument, namely that efficiency could be increased if AI could help triage and generate initial reports on a medical case (Table 3, Quote 2C). According to this group, the routine and repetitive tasks can be completed quickly, whereas writing a report is time-consuming and therefore worth allocating to an AI application.

In spite of the agreement with regard to the time-saving possibilities of AI, respondents differed in the degree to which they thought AI might support more complex diagnoses. Some believed that AI should eventually make automated decisions for straightforward diagnoses, while others said that AI should perform a certain pre-screening of a medical image and make suggestions for diagnosis. For more complex cases, pathological decision making requires the integration of information from various sources, and some respondents uttered the hope that AI may help to integrate these relevant sources of knowledge (Table 3, Quote 2D). Others expressed a hope that AI might become able to perform diagnostic tasks that are not (easily) performed by pathologists. Some of the respondents believed that AI could eventually be able to assist in detecting rare cases, which the average pathologist might miss due to lack of experience with those specific disease patterns. Others expressed a wish for AI that could provide prognostic analyses to determine if a patient would develop a (progressive) disease.

Some respondents also envisioned AI as a means of assessing images more consistently; this could help pathology adopt a more evidence-based approach that would be less dependent on individual execution (Table 3, Quote 2E). Furthermore, some pathologists wanted to learn from AI applications, especially if such tools could describe how they arrived at their decisions (Table 3, Quote 2F). This would mean that pathologists (in training) would no longer be solely reliant on the explanation and expertise of their supervising pathologist when learning how diagnoses are made. Similarly, a large number of respondents also fostered the hope that AI would help make pathological practices, which are inherently based on individual and expert interpretations, more standardized (Table 3, Quote 2G). Respondents who desired more standardization often cited the fact that an AI application, unlike a human expert, cannot tire and would always analyze a sample in the same way, uninfluenced by external factors (Table 3, Quote 2H). Nevertheless, some respondents suggested that a desire to be more objective was misplaced or even untenable (Table 3, Quote 2I). These respondents noted the fact that AI applications are developed and validated by drawing on expert opinion and can therefore never become truly objective. Still, these respondents admitted that such AI systems may foster discussions between pathologists and thereby indirectly lead to new perspectives or insights in specific cases – assuming it wouldn’t just further complicate already complex decision-making processes.

### Envisioned roles and responsibilities for AI

**Fig. 2:**
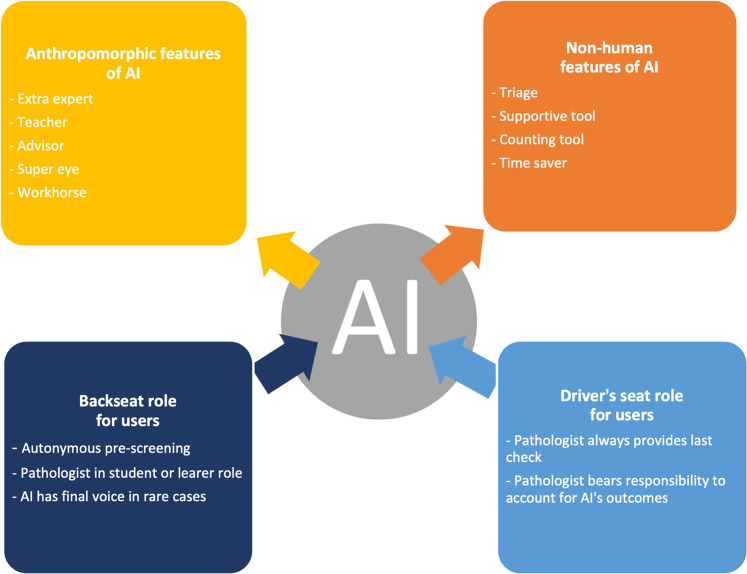
Envisioned roles for AI and end-users.

Respondents envisioned AI in a wide range of roles and respective responsibilities within the diagnostic process. Some of these have been mentioned already: AI could perform an autonomous pre-screening to support pathological decision-making processes, AI could overtake routine tasks (with or without final check of the pathologist), and AI could teach pathologists how it arrived at certain outcomes. The possible roles and responsibilities for AI according to respondents have been categorized in Fig. 2.

The envisioned roles and responsibilities for AI fall roughly into two categories: (1) those roles in which AI is ascribed anthropomorphic traits, and (2) those in which AI is seen as a non-humanlike technology. The first category focuses on the expert qualities of AI, where AI can take responsibility for (a part of) the diagnostic process and the health care professional is likely to take a backseat role when relying on AI outcomes. In this category, respondents described AI as becoming an extra expert in the diagnostic process, adding another view to the pathologist’s judgment, or AI as functioning as a *teacher* or advisor to the pathologist, proving information she does not have herself. Also, some respondents envisioned AI as a super eye or as being able to ‘see’ more in a digital image than human experts. Finally, respondents hoped to delegate simple, routine tasks to AI, describing it as a workhorse. The second category, on the other hand, describes roles in which AI might support pathologists in the same way as any other technology or tool. In these roles, pathologists would retain responsibility over the complete diagnostic process and actively assess algorithmic outcomes, taking on a driver-seat role as users. Possible supporting roles AI might adopt are as a triage, selecting possible cases where the pathologist’s judgment is required, or as a counting tool, for example counting mitoses in a tissue sample. Other general non-human like roles respondents mentioned were AI as a supportive tool to help pathologists analyze large data sets, and AI as a time saver, because it has the potential to make pathologists more efficient. These envisioned roles and responsibilities are not mutually exclusive; sometimes respondents advocated for several of these roles and were uncertain to what extent AI might indeed be able to take responsibility in the diagnostic process.

### Envisioned roles and responsibilities for pathologists

Most respondents assumed that pathologists would continue to be ultimately responsible for the diagnosis. Several respondents affirmed that while AI might take on many roles in supporting pathologists, they would feel comfortable overruling AI if necessary (Table 4, Quote 4A). Some described this metaphorically by comparing AI with an autopilot function in an airplane or machines in clinical chemistry: both the pilot and the clinical chemist must take responsibility for the machine’s outcomes and in the case that failures in the machine’s functioning occur (Table 4, Quote 4B and 4C).

Even though many respondents believed the responsibility for an AI-assisted diagnosis would likely remain with pathologists, they were simultaneously not (very) interested in understanding the inner workings of algorithms. When talking about the way they would use and interpret AI, pathologists indicated they did not think it would be necessary to know every step in an algorithm’s decision-making process as long as the outcomes could be validated and therefore trusted. Several respondents mentioned the fear pathologists and lay-people have concerning the black box nature of deep learning systems, but argued that reproducibility of AI outcomes is much more important for diagnostic purposes than understanding the black box itself (Table 4, Quote 4D). In other words, being able to check an algorithm’s performance and consistency would be sufficient. To illustrate this point, some compared AI to mobile phones and explained that one could use it correctly with just a basic understanding of the principles underlying its functioning (Table 4, Quote 4D and 4E). One of the computer scientists interviewed also pointed out that black box technologies are present in everyone’s daily lives, and we trust them even though we do not know what happens inside the technology (Table 4, Quote 4F). As multiple respondents noted, the current discussion on AI’s black boxes stems primarily from the fear of the unknown, rather than the need for a deep understanding of technical details.

Nevertheless, some respondents advocated for a middle ground somewhere between fully explainable AI and black box AI. Should AI be used for simple and routine tasks within pathology, it might be less important for pathologists to understand how it arrives at a certain conclusion. However, if AI would be used in the future to completely diagnose a complex disease or produce a prognosis, then it would be important to know more about how AI works (Table 4, Quote 4G). Also, if pathologists should be responsible for algorithmic failures, they should have at least a basic understanding of AI design (Table 4, Quote 4H). According to these respondents, the need for transparency and explainability should directly relate to the degree of responsibility AI would bear and the severity of the consequences in case of a diagnostic mistake.

## Discussion

Respondents provided a range of possible ways in which AI could be embedded within pathology. In this sense, they affirmed the assertion that AI holds a large promise to better the field. In the following discussion, their perspectives will be further contextualized amidst social and ethical literature, which has described several conditions for the implementation of medical AI^28–39^. By connecting the results of this study to broader challenges in AI implementation, we will also shed light on important insights which the participants in this study have provided. Gaining a better view of the way participants perceived the possibilities of AI, as well as important prerequisites or conditions, can assist future AI implementation. Specifically, the results of this study have pointed to three concrete recommendations for departments interested in integrating AI in a way that optimally aligns with end-users’ expectations and daily responsibilities.

### Recommendation 1: Foster a pragmatic attitude toward AI development

Contrary to much of the existing literature on the promises and pitfalls of medical AI^1–11,28–39^ and despite their belief in the promise of AI, respondents in this study showed a pragmatic attitude towards its actual implementation. Although some of the respondents did show idealistic tendencies when focusing on the future benefits of AI, they were also conscious of the problems facing implementation and focused on the practical usefulness of the technology for certain diagnostic tasks. Also, while they were generally open to the possibility that AI would become an integrated part of their standard workspace, they did not describe it as a wonder tool or silver bullet for problems related to efficiency or objectivity. This pragmatic stance toward AI is due, in part, to previous technological developments and “revolutions” in the field, which may have normalized the introduction of new technological changes^4,9,29,33,40^. It is also intrinsically related to the complex nature of pathologists’ work and the collaborative and multidisciplinary nature of most diagnostic processes.

This pragmatism can contribute to the responsible introduction of AI technologies, given that these highly depend on the interaction with skilled medical experts. As members of the pathology departments will likely take responsibility for AI outcomes used within the diagnostic process, it is essential for them to assess technological possibilities in a realistic light. The success of a technology such as AI is not only dependent on the accuracy with which it performs its functions, but on its fit with the medical and social context as well^41^. If pathologists would take responsibility for AI outcomes, AI would for instance need to be able to function in real contexts and with real-time data, and medical norms should be applicable to the way it analyses medical content. The significance of a good fit between AI and its end-users is often described as AI alignment or human-AI cooperation; these terms highlight the importance of aligning the values, needs and wishes of the practitioners with the technological design of AI^41,42^. The pragmatism of respondents therefore points to the necessary compatibility between AI design and the real social-medical contexts in which these will be implemented.

### Recommendation 2: Provide task-sensitive information and training to health care professionals working at pathology departments

The range of envisioned roles and responsibilities for AI in pathologists’ daily practices indicates that a wide variety of applications can potentially be developed or integrated. It also reveals that many members of pathology labs could and would like to learn more regarding the actual possibilities and limits of AI. Task-sensitive training should therefore be provided for all members of a department integrating AI. This training should include basic information about what AI is, how it works, the possibilities and limits of its applicability, what kinds of data it uses, and the types of metadata it generates. A shared understanding of what AI is and how it works can help establish the necessary support base to foster development and implementation that best aligns with the expectations and needs of the department.

Moreover, as our results emphasize, knowledge about computers and the inner workings of AI is often compared to ‘simple’ technologies such as a mobile phone, an autopilot function or black box. Although these comparisons can help describe the ways in which users interact with these technologies, they neglect or downplay the complexity of the technologies and may prevent a more nuanced understanding of their impact on daily practices^43^. Respondents indicated that they have a broad understanding of the knowledge they need to work and trust AI in their workplace, however many of them also emphasized the need to have a clearer idea of AI design if it were to be implemented in complex decision-making processes. It may therefore be important to focus more on providing nuanced knowledge on AI functioning so members of pathology departments can build their trust and feel confident taking responsibility for algorithm-supported diagnoses.

Interestingly though, respondents did not seem to always regard ‘explainability’ of the inner workings of AI as an essential requirement for AI integration, which implies that they seem to be less concerned about the opaque nature of AI than is suggested by certain discussions^44–48^. It is important to note here that the participants of this study have interpreted ‘explainability’ in a broad sense; namely, that specific reasons underlying an outcome could be made transparent. We acknowledge there are many definitions possible for explainability, and that specific use cases may demand kinds of explainability which were not discussed in this analysis. It may therefore be salient to further investigate how pathologists would view specific forms of explainability in various contexts. Within the context of this study in any case, respondents were more interested in gaining an understanding of AI’s underlying principles and hoped AI would introduce more standardized knowledge to pathology. This indicates an interest in furthering pathological knowledge by means of AI. Such progress in the state of knowledge can only be accomplished when professionals are equipped with task-sensitive knowledge on the way AI functions and AI tools are co-designed by pathologists, lab scientists and computer scientists representing diverse professional knowledge, values and standards^49,50^. An inclusive, departmentally wide approach towards future AI development and implementation can stimulate the sharing of expertise and work towards creating AI that truly represents a standardized account of pathological knowledge.

### Recommendation 3: Take time to reflect upon users’ changing roles and responsibilities

Lastly, the results of this study have further indicated that reflection upon users’ roles and responsibilities is necessary to attain a clearer idea of the changes members of pathology departments, especially pathologists, will undergo when AI is added to the diagnostic process. One danger of waiting for AI to be implemented before reflecting upon these issues is that the burden of AI – what will need to change in the department – will fall on the individuals who work with AI. This reflection is especially important, as many members must adjust simultaneously to the possibility of AI in their daily workflows and a recent transfer to working with digital systems. As research on computer use within other medical fields has emphasized, digitalization is not a value-neutral process and can create new power dynamics in which certain perspectives are more included in development and implementation processes than others^39,43^.

The future of AI in pathology is not a question of if it will be implemented, but when and how it will be implemented. The timing of and approach to the implementation are tantamount for successful integration of AI. On the one hand, some relatively simple AI tools used for, for instance, counting mitosis^26,51^ are already qualified to be implemented. Still, deliberation on their role within and impact on decision-making processes would be advisable. On the other hand, many AI tools are still being developed and, while the development process is on-going, it is essential to simultaneously think about the ways in which fundamental design choices affect AI’s roles and responsibilities^52^. Socio-technical challenges associated with the implementation of AI can benefit from early reflection, since anticipating upon future roles and responsibilities can prevent unwanted consequences for users. By establishing wanted and unwanted conditions for its implementation, possible unintended effects or even abuse may be detected^52^. This helps to retain decisional authority over the way AI is integrated, instead of ‘letting it just happen’^53^.

In order to generate effective reflection, it is important to adopt an open approach in which the adaptions to pathology generated by AI are scrutinized, such as can be seen in ethics parallel research or Value Senstive Design (VSD)^52,54^. As these approaches emphasize, it is tantamount to include reflection not just at one point in the development and implementation process, but to ask different normative questions at several stages of AI integration. This ensures that computational challenges such as agency, privacy and bias can be continuously targeted and values are intentionally embedded within a technology^52,54^. Reflection thus becomes an iterative process in which stakeholders think about the ways in which values are incorporated at several steps in the process of integration. Moreover, within the literature on AI development, there is an increasing call for inter- or multidisciplinary efforts to analyze value changes, as problems with algorithmic bias, trust and responsibility provide broader societal consequences^54^. We would therefore recommend reflection within pathology labs so (1) value changes are openly approached, (2) reflection is seen as an iterative process throughout the development and implementation processes and (3) is practiced at all levels (including patients and members other disciplines, where appropriate).

### Study limitations and recommendations for further research

In this study, we investigated the views of respondents who are working in pathology departments which have already adopted digital pathology. In doing so, we had limited access to the departments themselves due to the pandemic. Furthermore, there could be a potential loss in nuance by translating the interviews from Dutch to English. Although we reached saturation in the themes and codes identified, it would be interesting to include the perspectives of professionals who work at non-digital and non-academic pathology departments for comparison. This study included the perspectives of lab technicians and computer scientists; future studies might also consider focusing specifically on their professional roles in and attitudes towards future AI development. Because this study focused on the potential of AI for pathology, it did not go deeply into respondents’ interpretation of important concepts, such as what it means for AI to gain responsibility in the diagnostic process. It would be highly relevant to further investigate the ways in which normative concepts are given substance to by pathology professionals and what kind of normative frameworks can be developed to fit the wishes of medical practitioners.

## Concluding remarks

This study responded to the widely held belief that pathology, centered around image-based diagnostics, is one of the medical specializations most suited for implementing AI within the decision-making process^1–5^. The large number of images processed by pathology labs can be digitally uploaded and then analyzed by AI tools on several parameters for diagnosis^1^. This is confirmed by the large number of AI tools currently being developed to support pathologists in their diagnostic process. On paper, it is therefore mostly a ‘simple’ question of opportunity: When are the circumstances right for AI to be implemented in pathology? In reality, this question proves harder to answer; technical as well as ethical challenges to AI implementation have been formulated and require a clear strategy to tackle them^8,11,31^. Specifically, it requires members of pathology labs, with their extensive knowledge on practices, roles and responsibilities within pathology, to reflect on the way in which AI can and should be implemented in diagnostic process.

In order to gain insight in their perspectives, this article has provided the findings from the first in-depth interview study in which the expectations of pathologists, lab technicians and computer scientists on AI development and implementation are explicated. By discussing the future of AI within pathology, the participants have contributed to conceptualizing AI challenges by formulating the perceived possibilities of AI, as well as some important prerequisites or conditions that could be necessary for successful AI implementation. Specifically, the results of this study have pointed to three concrete recommendations for departments interested in integrating AI in a way that optimally aligns with end-users’ expectations and daily responsibilities. These recommendations are targeted at strengthening the compatibility of AI design and implementation processes with the social and medical norms guiding pathological practice.

As the literature also points out^52,54^, it is important to reflect on the ways in which technologies such as AI impact medical practice, also during the stages between development and implementation. Moving from the theoretical possibility of AI to practical implementation demands a change from members of pathology departments; this change can be guided, and concrete steps can be taken to make the change more manageable. Moreover, deciding over the *when*, as well as the if and how, of AI implementation requires a variety of perspectives and knowledge to enable AI to live up to its promises.

## Data Availability

The datasets used and analyzed during the current study are unavailable, as they are protected under the General Data Protection Regulation (GDPR: article 9).
