# Correction to: Integrating artificial intelligence in pathology: a qualitative interview study of users’ experiences and expectations

**DOI:** 10.1038/s41379-022-01164-x

**Published:** 2022-09-23

**Authors:** Jojanneke Drogt, Megan Milota, Shoko Vos, Annelien Bredenoord, Karin Jongsma

**Affiliations:** 1 Department of Medical Humanities, University Medical Center, Utrecht, The Netherlands; 2 Department of Pathology, Radboud University Medical Center, Nijmegen, The Netherlands

**Keywords:** Medical ethics, Medical imaging, Pathology

Correction to: *Modern Pathology* 10.1038/s41379-022-01123-6, published online 04 August 2022

After the publication of the original article the authors came across two important errors in the manuscript: (1) probably an autocorrect has changed the funding agency’s name from ‘’NWO’’ to ‘’NOW’’ (so the correct spelling is **NWO**) and (2) Sally Wyatt and Flora Lysen should be mentioned under ‘’contributions’’ with ‘**’We thank Sally Wyatt and Flora Lysen for their valuable comments on this work**.’’ The original article has been corrected.

